# Supplementary material for: Multiomics analysis reveals flavonoid accumulation and biosynthesis across different cultivation years and localities of Gongronemopsis tenacissima (Dai-Bai-Jie)
Source: PeerJ. 2025 Dec 16;13:e20439. doi: 10.7717/peerj.20439 (PMC12716135; doi:10.7717/peerj.20439)
Supplement: Supplemental Information 4 [file peerj-13-20439-s004.docx]

| Sample | | Shannon | Chao1 | ACE | Goods_coverage |
| --- | --- | --- | --- | --- | --- |
| 16s | CM1-1 | 10.199 | 4408.783 | 4510.324 | 0.976 |
|  | CM1-2 | 10.198 | 4408.120 | 4455.396 | 0.976 |
|  | CM1-3 | 10.428 | 5717.255 | 5770.891 | 0.965 |
|  | CM2-1 | 9.493 | 4549.834 | 4731.459 | 0.971 |
|  | CM2-2 | 9.692 | 4249.906 | 4264.581 | 0.975 |
|  | CM2-3 | 9.454 | 4549.623 | 4694.692 | 0.971 |
|  | CM3-1 | 9.341 | 4458.525 | 4582.019 | 0.972 |
|  | CM3-2 | 9.524 | 4704.325 | 4812.714 | 0.970 |
|  | CM3-3 | 9.657 | 4578.785 | 4694.049 | 0.973 |
|  | CM4-1 | 10.086 | 4800.169 | 4856.335 | 0.972 |
|  | CM4-2 | 9.962 | 4743.262 | 4827.648 | 0.971 |
|  | CM4-3 | 10.07 | 5027.961 | 5124.025 | 0.969 |
| ITS | CM1-1 | 5.833 | 1234.177 | 1238.847 | 0.997 |
|  | CM1-2 | 5.612 | 1138.532 | 1164.348 | 0.997 |
|  | CM1-3 | 6.237 | 1249.585 | 1280.700 | 0.997 |
|  | CM2-1 | 5.184 | 1069.941 | 1098.892 | 0.997 |
|  | CM2-2 | 4.856 | 945.519 | 974.251 | 0.998 |
|  | CM2-3 | 5.762 | 1443.363 | 1484.480 | 0.996 |
|  | CM3-1 | 6.038 | 1490.137 | 1531.811 | 0.996 |
|  | CM3-2 | 5.593 | 1524.667 | 1549.868 | 0.995 |
|  | CM3-3 | 5.534 | 1211.356 | 1255.569 | 0.997 |
|  | CM4-1 | 6.301 | 1440.550 | 1468.119 | 0.996 |
|  | CM4-2 | 6.198 | 1347.688 | 1360.925 | 0.997 |
|  | CM4-3 | 6.462 | 1486.053 | 1518.814 | 0.996 |
